# α-Synuclein increases β-amyloid secretion by promoting β-/γ-secretase processing of APP

**DOI:** 10.1371/journal.pone.0171925

**Published:** 2017-02-10

**Authors:** Hazel L. Roberts, Bernard L. Schneider, David R. Brown

**Affiliations:** 1 Department of Biology & Biochemistry, University of Bath, Claverton Down, Bath, United Kingdom; 2 Brain Mind Institute, Ecole Polytechnique Fédérale de Lausanne (EPFL), Lausanne, Switzerland; USF Health Morsani College of Medicine, UNITED STATES

## Abstract

α-Synuclein misfolding and aggregation is often accompanied by β-amyloid deposition in some neurodegenerative diseases. We hypothesised that α-synuclein promotes β-amyloid production from APP. β-Amyloid levels and APP amyloidogenic processing were investigated in neuronal cell lines stably overexpressing wildtype and mutant α-synuclein. γ-Secretase activity and β-secretase expression were also measured. We show that α-synuclein expression induces β-amyloid secretion and amyloidogenic processing of APP in neuronal cell lines. Certain mutations of α-synuclein potentiate APP amyloidogenic processing. γ-Secretase activity was not enhanced by wildtype α-synuclein expression, however β-secretase protein levels were induced. Furthermore, a correlation between α-synuclein and β-secretase protein was seen in rat brain striata. Iron chelation abolishes the effect of α-synuclein on neuronal cell β-amyloid secretion, whereas overexpression of the ferrireductase enzyme Steap3 is robustly pro-amyloidogenic. We propose that α-synuclein promotes β-amyloid formation by modulating β-cleavage of APP, and that this is potentially mediated by the levels of reduced iron and oxidative stress.

## Introduction

Protein misfolding is an integral feature of age-related neurodegenerative diseases, and stereotypically involves just a handful of conformationally flexible proteins. Parkinson’s disease (PD) and Dementia with Lewy bodies (DLB) are defined by intracellular inclusions of aggregated α-synuclein, known as ‘Lewy bodies’ [1]. Alzheimer’s disease (AD) brains, in contrast, predominantly exhibit extracellular amyloid plaques of β-amyloid, and intracellular inclusions of tau protein [2–5]. Yet a sizeable proportion of patients classified as DLB or AD have both α-synuclein and β-amyloid aggregates [6–14], leading to the suggestion that the two proteins may be somehow linked [15]. The connection is likely to have a perceptible effect on disease progression. Biomarkers for β-amyloid deposition, but not tau aggregation, appear to predict the incidence of dementia in PD patients, and correlate with dementia severity [16–21]. Approximately 30% of PD patients have dementia, which may rise to 70–80% within a decade of PD diagnosis [22].

The idea that α-synuclein and β-amyloid may directly or indirectly interact is not new. Previously α-synuclein and β-amyloid have been shown to have synergistic toxicity in cells and transgenic mice [23–25], and may promote one another’s aggregation in vitro [24,26–29]. Co-aggregation or ‘cross-seeding’ of α-synuclein and β-amyloid to create heterogenous oligomers/fibrils is not supported by recent mouse studies. In vivo, α-synuclein appears to inhibit the formation of β-amyloid fibrils [30,31]. Yet there are indications that α-synuclein may also influence β-amyloid in another way. Treatment of cells with recombinant α-synuclein causes β-amyloid levels to increase [32,33]. The molecular mechanism was not fully explored, but has been proposed to involve phosphatidylinositol 3-kinase (PI3K) signalling [33]. The generation of β-amyloid from its precursor, the amyloid precursor protein (APP), is an important regulatory step. During amyloidogenic processing, APP is cleaved by β-secretase to shed a soluble extracellular N-terminal domain. The remaining C-terminal fragment (C99) is cleaved by γ-secretase to release a β-amyloid peptide (Aβ) and an intracellular domain (AICD). The specific changes to amyloidogenic processing caused by α-synuclein have not been previously investigated.

Regulation of β-amyloid production is complex, factoring in secretase expression, subcellular localisation, and competition from other APP processing enzymes [34]. Yet these regulatory processes frequently respond to a common upstream stimulus, one example of which is oxidative stress. Oxidative stress has been reported to upregulate β- and γ-secretase transcription, translation of the β-secretase, and β-secretase co-localisation with APP [35–41]. Amyloidogenic processing is also upregulated by free iron, which can generate reactive oxygen species (ROS) through Fenton reactions [42–44]. α-Synuclein is a putative ferrireductase, and therefore a potential source of free reduced iron [45,46]. Increased generation of mitochondrial-derived ROS is also observed in α-synuclein-overexpressing models [47].

In this paper we use a stable overexpression cell model of α-synuclein to confirm that it increases secreted β-amyloid levels. Furthermore we characterised underlying changes to β-amyloid production from APP, by luciferase reporter assays and the measurement of an intermediate APP metabolite, C99. β-Secretase expression and γ-secretase activity were also measured. A number of mutant α-synuclein lines were tested to ascertain whether loss-of-function or gain-of-toxicity of α-synuclein could be linked to altered APP processing. Finally, we present evidence that supports a potential role for iron-mediated oxidative stress in changes to APP amyloidogenic processing induced by α-synuclein. The overall aims of this work were to characterise amyloidogenic processing of APP in cell models of synucleinopathy, and to find evidence of the underlying cell mechanism. Successful characterisation of APP processing was achieved in multiple synucleinopathy cell models, confirming the potential importance of α-synuclein to β-amyloid production, however the underlying cell mechanism is tentative.

## Materials and methods

### Compounds

TAPI-1, Amyloid Precursor Protein β-Secretase Inhibitor (βSI), and β-Secretase Inhibitor IV (βIV) were purchased from Merck Millipore. DAPT was purchased from Tocris. TAPI, βSI, βIV, and DAPT were made up as 1000x concentrated stocks in DMSO (Sigma).

### DNA constructs

Plasmids encoding wildtype α-synuclein (RefSeq accession number XM_011532204) and β-synuclein (RefSeq accession number NM_001001502) in a pcDNA 3.1 (+) vector were previously described [48,49]. A pCI-neo-APP695 plasmid was kindly donated by Prof. Chris Miller, Kings College London [50]. Steap3 plasmid was generated by cloning the human Steap3 sequence (RefSeq accession number NG_042823.1) into a pcDNA 3.1 (+) vector. pFR-Luciferase reporter vector (pLuc) containing firefly (*Photinus pyralis*) luciferase gene, with a synthetic promoter of yeast Gal4 upstream activation sequence in 5 tandem repeats upstream of a minimal TATA box, was from Promega. phRL thymidine kinase vector (pTK) containing sea pansy (*Renilla reniformis*) luciferase gene, under control of the herpes simplex virus-TK promoter, was also from Promega. pRC-CMV-APP695-Gal4 (APP-Gal4) and pSec-Tag2-Notch3Gal4 (Notch-Gal4) were kindly provided by Dr Robert J. Williams, University of Bath [51,52]. Human BACE1 promoter luciferase reporter, a pGL3-Basic vector containing a 4.3kb fragment of BACE1 promoter from -4372 to -1 of the promoter sequence, was previously described [53].

### Cell cultures and transfection

SH-SY5Y neuroblastoma cells (obtained directly from ATCC, cat no. CRL-2266) were grown in 1:1 DMEM (high glucose with L-Glutamine, Lonza) and Ham’s F-12 (Lonza), supplemented with 10% fetal bovine serum (Sigma), 100 U/mL penicillin, and 100 μg/mL streptomycin (Sigma). Growth conditions were maintained at 37°C and 5% CO_2_ in a humidified incubator. Cells were stably transfected with pcDNA 3.1 (+)-α-synuclein, or mutations thereof. The mutants included truncations Δ2–9, at the extreme N-terminus, and Δ71–82, in the NAC domain of α-synuclein. PD-associated point mutations A30P, E46K and A53T were also included. Additionally two substitutions of S129, a phosphorylation site in the C-terminus of α-synuclein. S129A blocks phosphorylation, whereas S129D aims to mimic permanent phosphorylation. Transfection was achieved using FuGene HD lipid reagent (Promega) according to the manufacturer’s instructions. Stable selection was performed with 0.8 mg/ml G418 (Sigma) 24 hours after transfection, and cells maintained in 0.4 mg/ml G418. Successful transfections were assessed by western blotting to ensure over-expression of the protein.

### Antibodies

Rabbit monoclonal anti-α-synuclein (MJFR1, Abcam, immunogen human α-synuclein 1–150) was used for human α-synuclein detection at a dilution of 1:4000. Mouse monoclonal anti-α-synuclein (610787, BD Biosciences, immunogen rat α-synuclein 15–123) was used for rat α-synuclein detection at a dilution of 1:2000. Mouse monoclonal anti-α-tubulin (T5186, Sigma, immunogen acetylated tubulin from *Strongylocentrotus purpuratus* sperm axonemes) was used at a dilution of 1:10,000. Rabbit monoclonal anti-APP C-terminus (Y188, Abcam immunogen human APP750 C-terminus) was used at a dilution of 1:2000. Rabbit monoclonal anti-BACE1 (D10E5, Cell Signaling Technology immunogen human BACE1 residues around His490) was used at a dilution of 1:1500.

### Western blotting

Cells were lysed in 0.5% Igepal CA-630 and ‘complete’ protease inhibitor cocktail (Roche), sonicated 3 x 3 seconds on ice, and centrifuged 10 000 xg for 3 minutes to remove insoluble membranes. Protein concentration was determined with a Bradford protein assay (Bio-Rad), according to the manufacturer’s instructions. Supernatant protein concentrations were normalized and boiled for 5 minutes with 1 x Laemmli SDS-PAGE buffer. To determine levels of α-synuclein, full-length APP, or BACE1: samples were loaded into a 12% acrylamide SDS-PAGE gel, with a buffer of Tris (250 mM) + Glycine (1.92 M) + SDS (0.1% w/v), run at 250V for 45 minutes. To resolve bands of C99 and C83 APP: samples were loaded into a 16% acrylamide + 10% glycerol SDS-PAGE gel, with an anode buffer of Tris-HCl (20 mM, pH 8.9), and a cathode buffer of Tris (100 mM) + Tricine (100 mM) + SDS (1% w/v). The 16% gel was electrophoresed at 100 V, 4°C, for several hours. Separated proteins were transferred to a PVDF membrane by a semi-dry transfer apparatus, run at 25V for 1.3 hours. Membranes were blocked in 5% w/v non-fat milk powder dissolved in TBS-T (0.05% Tween-20, 10 mM Tris, 100 mM NaCl) for 30 minutes, incubated with primary antibody for 1–2 hours, and washed 3 x 5 minutes in TBS-T. Membranes were blocked again and incubated with horseradish peroxidase-conjugated secondary antibody for 1 hour. A further 3 x 10 minute washes were performed, and the membranes developed with Luminata Crescendo or Luminata Forte ECL substrate (Thermo Scientific), and imaged with a Fusion SL CCD imaging system (Vilber Lourmat).

### Measurement of Aβ40 and Aβ42 by Meso Scale Discovery assay

Fresh serum-free B-27-supplemented DMEM, ± compounds, was added to SH-SY5Ys a day after seeding in 24-well plates. Conditioned media was collected after 72 hours and immediately assayed without further manipulation, using the V-PLEX Plus Aβ Peptide Panel 1 (6E10) Kit from Meso Scale Discovery, according to the manufacturer’s instructions. The plate was read with a Sector Imager 6000 (Meso Scale Discovery). Peptide concentrations (pg/ml) were calculated by Meso Scale Discovery Workbench software, with reference to a standard curve, and were normalised to the mean concentration for each experiment.

### Dual glo luciferase reporter assay

SH-SY5Ys in 24-well plates were co-transfected with plasmids complexed with 1.5 μl/well FuGene HD (Promega). For the APP-Gal4 reporter assay for amyloidogenic processing, cells were transfected with 50 ng each of APP-Gal4 plasmid, pLuc, and pTK. For measuring human BACE1 promoter activity, cells were transfected with 200 ng of BACE1 promoter luciferase reporter plasmid, and 50 ng pTK. For the Notch-Gal4 reporter assay for γ-secretase activity, cells were transfected with 100 ng of Notch-Gal4 plasmid, and 50 ng each of pLuc and pTK. Luciferase activity was measured 20–22 hours post-transfection using the Promega Dual-Luciferase Reporter Kit according to the manufacturer’s instructions. Raw data was normalised by division with the mean firefly or Renilla luminescence for that experiment. Relative Luciferase Units (RLU) were calculated for each well by division of the firefly signal by the *Renilla* signal. The average RLU for 3–5 replicate transfections was calculated in each experiment.

### Oxidative stress assay

Cells were seeded at a density of approximately 1x10^6^ cells/ml onto a poly-D-lysine-coated 48-well plate. After 48 hours, cells were incubated with 10 μM CM-H_2_DCFDA probe in HEPES-buffered media (20 mM HEPES, 140 mM NaCl, 5 mM KCl, 5 mM NaHCO_3_, 1.2 mM Na_2_HPO_4_, 1.2 mM CaCl_2_, 5.5 mM glucose) for 20 minutes. The probe was removed and 300 μl of HEPES-buffered media added. Fluorescence intensity (Ex/Em = 488/534 nm) was measured every 5–10 minutes for 60 minutes. The linear rate equation was determined from a kinetics plot using MS Excel, and the rate at 60 minutes calculated. For each experiment, rates were normalised to the average.

### Viral vector production and titration

Serotype 6 adeno-associated viral (AAV6) vectors were produced and titrated as previously described [54]. The number of transducing units (TU) was determined by infecting HEK293T cells. The number of S1-nuclease resistant vector genome copies was measured by real-time PCR at 48 hrs post-infection. The AAV6-α-syn vector encodes expression of full-length wild-type human α-syn under the control of the constitutive mouse pgk-1 promoter. The titre of the vector suspension was 7.9E10 TU/ml.

### Animal experiments

All procedures were performed in accordance with Swiss legislation and the European Community Council directive (86/609/EEC) for the care and use of laboratory animals. Ethical Approval was obtained from the Committee on Animal Experimentation of the Canton de Vaud, Lausanne, Switzerland (permit number 1653.4). The experiments were carried out so as to minimize the suffering of the animals and to comply with the 3R principles. For biochemical analysis, the animals were culled by decapitation and fresh brain tissue immediately collected and snap frozen. Female adult Sprague-Dawley rats (Charles River Laboratories, France), weighing 180–200 g, were housed in a 12-hour light-dark cycle, with *ad libitum* access to water and food. Rats were injected with empty or α-synuclein-encoding AAV6 vectors in the right *substantia nigra*, with the non-injected left acting as an internal control. The total injected dose for each animal was 2.8E7 TU in a volume of 2 μl. The general procedure for vector injection was as previously described [54]. We used the following stereotaxic coordinates: 5.2 mm anterior and 1.9 mm lateral to bregma point, 7.9 mm ventral from the skull surface. The injected rats were culled one month after injection. For biochemical analysis, fresh striatal tissue was obtained from each brain hemisphere separately. Neuropathology and motor deficits of the rat model have been previously characterised [54–56]. Additionally, brain α-synuclein expression and aggregation were visualised by immunohistochemistry. For immunohistochemistry, the rats were sacrificed three months post-injection, and the brains were perfused with 4% paraformaldehyde (1.5 h post-fixation), before being transferred to 25% sucrose solution. Immunostaining for tyrosine hydroxylase (Millipore, #AB152; 1:1000), total α-syn (Millipore #AB5334P; 1:1000) and aggregated α-syn (clone 5G4, Millipore #MABN389; 1:1000) were performed on striatal sections (25 μm) from the rat striatum and substantia nigra, using standard procedures.

### Statistical analysis

Statistical analysis was performed using MS Excel. With the exception of the rat material, all data were analysed by unpaired two-tailed Student’s t-tests, with an assumption of equal variance. Rat striata were analysed by a paired two-tailed Student’s t-test. Differences were defined as statistically significant when p < 0.05.

## Results

### α-Synuclein and APP protein expression are not interlinked

**Fig 1:**
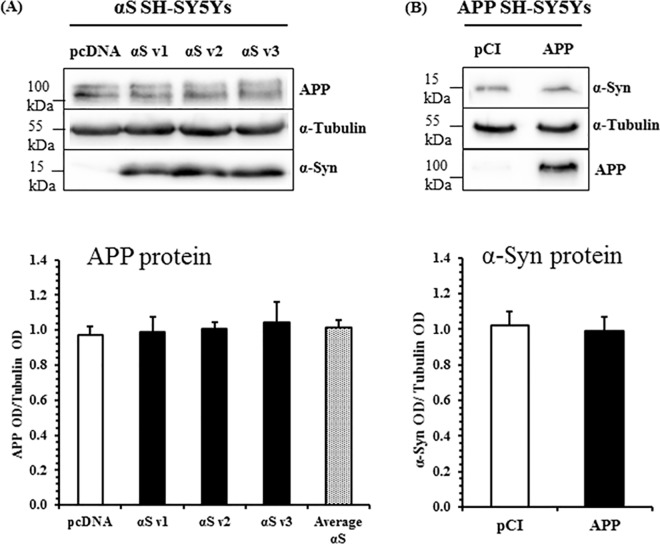
α-Synuclein and APP Protein Expression Are Not Interlinked. (A) APP protein in α-synuclein overexpressing SH-SY5Ys. Data for three independently generated polyclonal lines is shown, denoted ‘αS v1’, ‘αS v2’, and ‘αS v3’. Mean ± S.E. of 4 independent experiments. (B) α-Synuclein protein in APP overexpressing SH-SY5Ys. Raw band optical intensities (OD) were normalised to the mean OD of the experiment by division, and expressed as a ratio of the test protein to α-tubulin. Mean ± S.E. of 12 independent experiments. No significant differences, Student’s t-test.

The possibility that α-synuclein and APP may alter one another’s expression was first addressed using SH-SY5Y human neuroblastoma cells, stably overexpressing either protein. Three separate cell lines overexpressing α-synuclein were generated (v1-3). Over-expression was confirmed using western blot (S1 Fig). Relative levels of full-length APP protein in α-synuclein overexpressing SH-SY5Y lines were determined by western blotting (Fig 1*A*). No changes to APP expression were detected in comparison to pcDNA cells. APP695 was additionally overexpressed in SH-SY5Ys, to ascertain the effect on α-synuclein protein. α-Synuclein protein levels were unaltered (Fig 1*B*).

### α-Synuclein overexpression promotes β-amyloid secretion and β- / γ-secretase-mediated processing of APP

**Fig 2:**
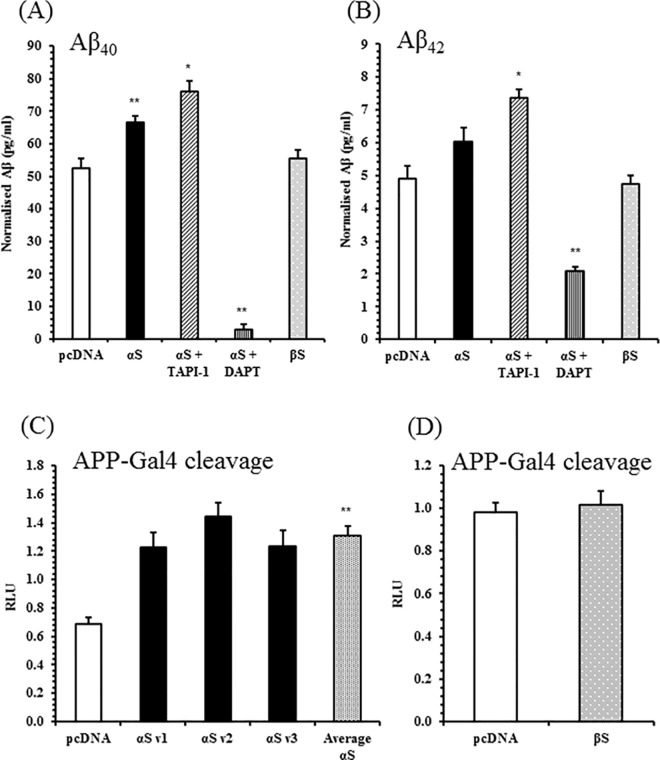
Increased β-Amyloid Production and APP Amyloidogenic Processing in α-Synuclein Overexpressing SH-SY5Ys. Levels of secreted Aβ40 *(A)* and Aβ42 *(B)* in conditioned media from 72 hours of culture were detected with the V-PLEX Plus Aβ Peptide Panel 1 (6E10) Kit from Meso Scale Discovery. α-Synuclein SH-SY5Ys (αS), and β-synuclein SH-SY5Ys (βS) were tested, and β-amyloid secretion shown to be sensitive to treatment with α-secretase inhibitor TAPI-1 (50 μM) and γ-secretase inhibitor DAPT (10 μM). Mean ± S.E. of 3–4 biological replicates across 3 independent experiments. APP-Gal4 activity in *(C)* three α-synuclein SH-SY5Y lines, *(D)* β-synuclein SH-SY5Ys. Mean ± S.E. of 5 independent experiments. * p < 0.05, ** p < 0.01 relative to pcDNA; Student’s t-tests. RLU: Relative Luciferase Units.

The amyloidogenic processing of APP was investigated in α-synuclein overexpressing SH-SY5Y cells (α-syn cells). β-Amyloid production was directly measured by a multiplex assay for Aβ40 and Aβ42 peptides secreted into medium of α-syn cells (Fig 2*A and* 2*B*). Compared to the control cells, Aβ40 accumulated significantly in the media of α-syn cells, Aβ42 did not. However, when comparing the mean Aβ40: Aβ42 ratio between control cells (11.3 ±0.7 SE) and α-syn cells (11.5 ±0.4 SE), no significant difference was found (p = 0.8, Student’s t-test). For comparison, a line overexpressing β-synuclein was also used. Expression of β-synuclein did not affect Aβ40 and Aβ42 levels. The dependence of extracellular β-amyloid accumulation on secretase-mediated processing was determined with secretase inhibitors. DAPT treatment inhibits γ-secretase, and strongly reduced extracellular Aβ40 and Aβ42 levels in α-syn cells. TAPI-1 inhibits the non-amyloidogenic α-secretase cleavage of APP, and enhanced Aβ40 and Aβ42 accumulation in α-syn cells. Control cells also saw a significant increase in Aβ40 and Aβ42 levels with TAPI-1 treatment (data not shown).

In addition to β-amyloid peptides, the β-/ γ-secretase-mediated cleavage of APP liberates an N-terminal ectodomain, and the APP intracellular domain (AICD). AICD production can be indirectly measured through a APP-Gal4 luciferase reporter system [51,52]. The APP-Gal4 assay preferentially reports β-/ γ-secretase processing in SH-SY5Ys, over α-/ γ-secretase processing (S2 Fig). APP-Gal4 reporter activity was measured in three lines of α-syn cells (Fig 2C), and appeared significantly increased by α-synuclein overexpression. Expression of β-synuclein did not alter APP-Gal4 cleavage (Fig 2*D*). To confirm that the effect of α-synuclein over-expression upon APP processing was not cell type specific, another neuronal cell line N2A was stably transfected with α-synuclein (S3 Fig) and tested for APP-Gal4 reporter activity (S4 Fig). APP-Gal4 cleavage was significantly increased in α-synuclein N2As, compared with pcDNA N2A cells. These results are consistent with an increase in amyloidogenic processing in cells overexpressing α-synuclein.

### α-Synuclein overexpression alters β-secretase but not γ-secretase activity in SH-SY5Ys

**Fig 3:**
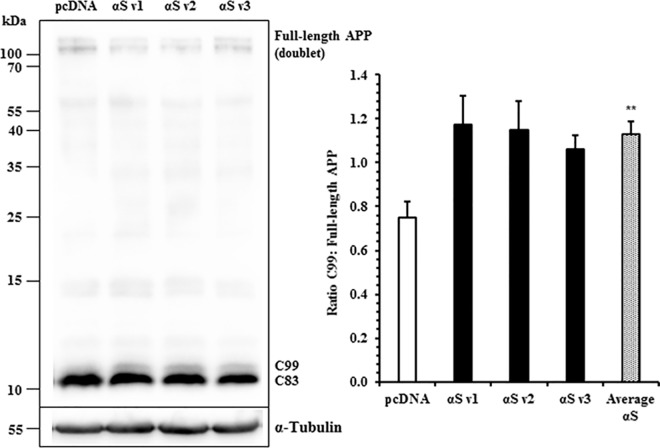
β-Cleaved C99 APP Production is Elevated in Wildtype α-Synuclein Overexpressing SH-SY5Ys. Levels of full-length, C99, and C83 APP were determined semi-quantitatively by western blotting. Raw band optical intensities (OD) were normalised by division with the mean OD of the experiment, and expressed as a ratio of C99 APP (~12 kDa) to full-length APP (~100 kDa). Mean ± S.E. of 4 independent experiments. **, p < 0.01; Student t-test.

Amyloidogenic processing may be promoted by an increase in β-secretase or γ-secretase activity, or by their increased co-localisation with APP. One way to specifically measure endogenous β-secretase-mediated cleavage of APP is by measuring C99, the C-terminal fragment (CTF) produced by β-cleavage. On western blots C99 can be resolved from C83, the CTF formed from α-secretase-mediated cleavage. To accumulate detectable levels of C99, cells were pre-incubated with γ-secretase inhibitor (S5 Fig). Protein extracts were electrophoresed by 16% SDS-PAGE and immunoblotted with a C-terminal APP antibody (Fig 3). In α-syn cells, levels of C99 were increased by an average of 60%, as a proportion of full-length APP.

**Fig 4:**
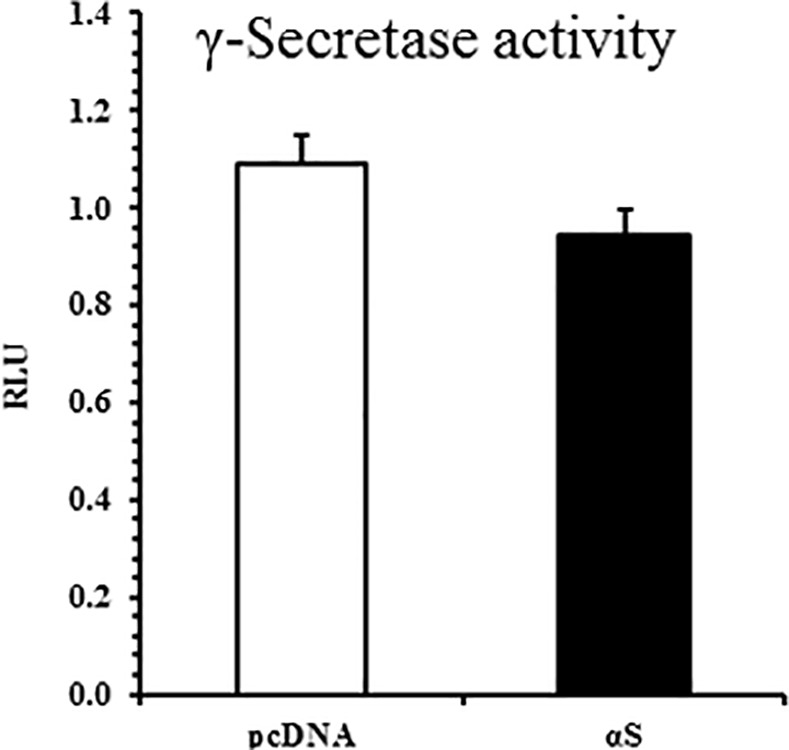
γ-Secretase Activity is Not Enhanced in α-Synuclein Overexpressing SH-SY5Ys. Notch-Gal4 luciferase reporter activity measured in αS v1 SH-SY5Ys. Mean ± S.E. of 8 independent experiments. No significant difference, Student’s t-test. RLU: Relative Luciferase Units.

γ-Secretase activity was also measured in α-syn cells. The Notch-Gal4 luciferase reporter for γ-secretase-mediated Notch cleavage was used to assess endogenous γ-secretase activity. γ-Secretase cleavage of Notch follows the shedding of the Notch N-terminal ectodomain by ‘α-secretases’ ADAM17 or ADAM10. Although γ-secretase inhibitor treatment impedes Notch-Gal4 cleavage in SH-SY5Ys, α-secretase inhibitor treatment did not alter Notch-Gal4 activity (S2 Fig). Measurements of γ-secretase activity in α-syn cells (αS v1) reveal no difference compared with pcDNA (Fig 4).

### BACE1 protein expression is post-transcriptionally upregulated in α-synuclein SH-SY5Ys

**Fig 5:**
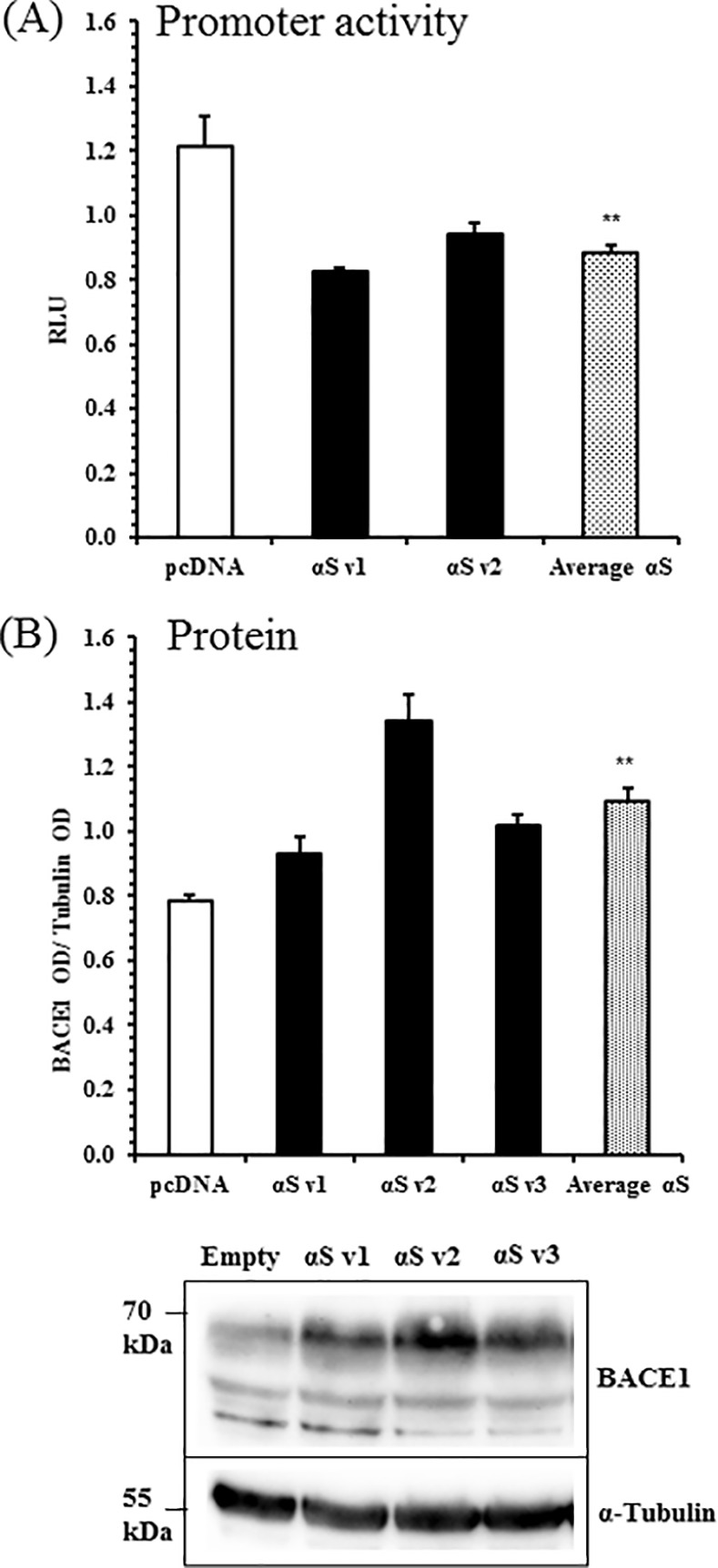
BACE1 Transcription is Reduced, but Protein Levels are Elevated, in Wildtype α-Synuclein Overexpressing SH-SY5Ys. (A) Human BACE1 promoter luciferase reporter assay in wildtype α-synuclein SH-SY5Ys. Mean ± S.E. of 5 independent experiments. (B) Whole cell lysates were tested for BACE1 and α-tubulin by western blotting. Raw band optical intensities (OD) were normalised by division with the mean OD of the experiment, and expressed as a ratio of BACE1 to α-tubulin. Mean ± S.E. of 5 independent experiments. *, p < 0.05; **, p < 0.01; Student t-tests.

β-Secretase cleavage of APP is performed by BACE1, and can be regulated either via control of BACE1 protein levels or co-localisation with APP. Transcriptional activity of the BACE1 promoter was determined in α-syn cells by a luciferase reporter. Transiently expressed in cells, the reporter construct contained a luciferase gene driven by a 4.3 kb fragment of the human BACE1 promoter [53]. BACE1 transcriptional activity was significantly reduced in α-syn cells relative to pcDNA (Fig 5*A*). BACE1 protein levels were also measured by western blotting. In contrast to the low transcriptional activity, total BACE1 protein levels were significantly enhanced in α-syn cells (Fig 5*B*).

### BACE1 expression is upregulated by α-synuclein overexpression *in vivo*

**Fig 6:**
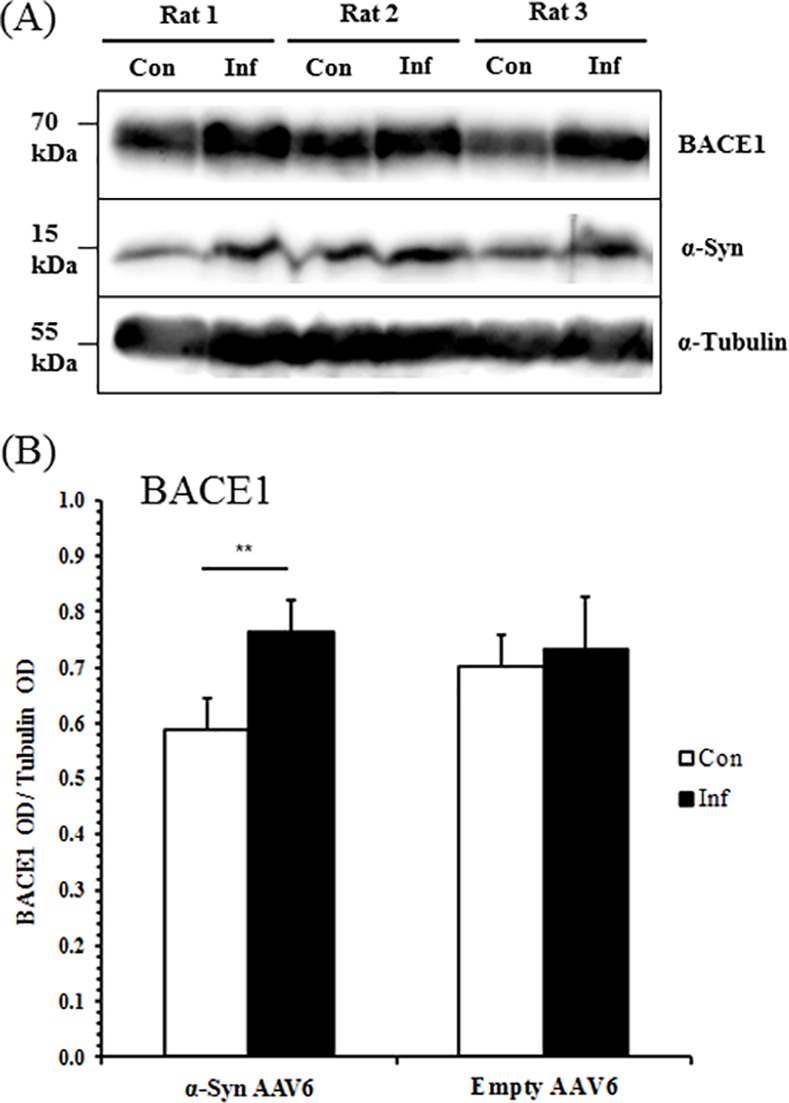
BACE1 Levels Correlate with α-Synuclein Expression in Rat Striata. Striata were obtained from rats unilaterally injected with AAV6 vector into the substantia nigra, both contralateral and ipsilateral to the hemisphere of injection. Homogenates were tested for BACE1, α-synuclein, and α-tubulin by western blotting. (A) Representative western blot image of BACE1 expression in striata from three α-syn AAV6-injected rats, contralateral (“Con”) and ipsilateral (“Inf”) to vector injection. (B) Rat striatum BACE1 total protein levels in α-syn AAV6- and empty AAV6-injected animals, comparing the hemispheres contralateral (“Con”) and ipsilateral (“Inf”) to vector injection. Mean ± S.E. of 8 animals injected with α-syn AAV6 and 4 animals injected with empty AAV6. **, p < 0.01; paired two-tailed Student’s t-test.

To establish whether α-synuclein and BACE1 expression are linked *in vivo*, immunoblotting was performed on rat brain. Rats were infected with an α-synuclein-encoding AAV6 vector by unilateral stereotaxic injection into the substantia nigra (SN). Striatum samples were obtained from both injected and non-injected hemispheres, and tested by western blotting. In this model system, overexpression of human α-synuclein and aggregation of the α-synuclein protein can be detected both in the substantia nigra and striatum, in the hemisphere ipsilateral to the site of vector injection (S6 Fig). The accumulation of human α-synuclein leads to progressive degeneration of the dopaminergic neurons and axons in the nigrostriatal system (S6 Fig). Transduction of the nigrostriatal system with α-synuclein was associated with significantly elevated BACE1 levels, relative to the untransduced control striata (Fig 6*A and* 6*B*). Rats injected with empty AAV6 vector did not exhibit a significant change in BACE1 expression (Fig 6*B*, S7 Fig).

### Mutants of α-synuclein reveal that APP processing is enhanced by disruption of the α-synuclein N-terminal domain

Additional SH-SY5Y cell lines overexpressing mutant α-synuclein were also utilised, to better understand the structural basis by which α-synuclein exerts an effect on APP processing. Overexpression of α-synuclein in the mutant lines was comparable to the wildtype line αS v1 (S8 Fig). As performed with the wildtype lines, mutant α-synuclein lines were tested for levels of secreted β-amyloid, amyloidogenic processing of APP, γ-secretase activity, BACE1 promoter activity, and levels of BACE1 protein (Table 1). Two mutants significantly potentiated the extracellular accumulation of Aβ_42_ and Aβ_40_: Δ2–9 and E46K. Interestingly Δ2–9 had the greatest effect, tripling β-amyloid levels. Underlying this was an eight-fold increase in APP-Gal4 cleavage and, uniquely, both γ-secretase activity and BACE1 transcription were elevated in Δ2–9 cells. BACE1 protein levels were also increased in Δ2–9 cells. E46K increased β-amyloid levels by 25%, with a similarly modest rise in BACE1 protein levels, and little impact on amyloidogenic processing (not statistically significant).

**Table 1:** Mutant α-Synuclein SH-SY5Ys.

SH-SY5Y line	Aβ40 (pg/ml *± SE*)	Aβ42 (pg/ml *± SE*)	APP-Gal4 cleavage (RLU *± SE*)	Notch-Gal4 cleavage (RLU *± SE*)	BACE1 promoter activity (RLU *± SE*)	BACE1 protein (relative band intensity *± SE*)
**pcDNA**	53 *± 3*	5 *± 0*.*4*	0.9 *± 0*.*0*	1.1 *± 0*.*1*	1.1 *± 0*.*0*	0.7 *± 0*.*0*
**αS v1**	67 *± 2*	6 *± 0*.*4*	1.31 *± 0*.*1*	0.9 *± 0*.*1*	0.9 *± 0*.*0*	0.9 *± 0*.*0*
**Δ2–9**	219 *± 13* **	20 *± 0*.*7* **	8.0 *± 1*.*8* **	4.2 *± 0*.*4* **	2.7 *± 0*.*2* **	1.5 *± 0*.*1* **
**Δ71–82**	70 *± 3*	6 *± 0*.*3*	3.7 *± 0*.*7* **	1.2 *± 0*.*1*	1.6 *± 0*.*2* *	1.1 *± 0*.*1* *
**A30P**	46 *± 1*	4 *± 0*.*1*	2.3 *± 0*.*5*	1.1 *± 0*.*1*	0.9 *± 0*.*1*	1.0 *± 0*.*1*
**E46K**	93 *± 3* **	8 *± 0*.*2* **	1.6 *± 0*.*3*	1.2 *± 0*.*1*	0.9 *± 0*.*1*	1.2 *± 0*.*1* *
**A53T**	61 *± 5*	5 *± 0*.*4*	2.0 *± 0*.*2* **	1.5 *± 0*.*2*	1.0 *± 0*.*1*	1.1 *± 0*.*1*
**S129A**	-	-	0.7 *± 0*.*1* * *#*	-	-	1.1 *± 0*.*1* *
**S129D**	-	-	1.5 *± 0*.*3* #	-	-	1.0 *± 0*.*1* *

Data displayed from SH-SY5Ys stably transfected with plasmids of Δ2–9, Δ71–82, A30P, E46K, A53T, S129A, and S129D α-synuclein: Aβ40 and Aβ42 levels in conditioned media were detected with the V-PLEX Plus Aβ Peptide Panel 1 (6E10) Kit from Meso Scale Discovery. Mean peptide concentration ± S.E. of 3–4 biological replicates across 3 independent experiments. APP-Gal4 cleavage luciferase reporter assay provides a measure of amyloidogenic processing. Mean RLU ± S.E. of 3–5 independent experiments. Notch-Gal4 cleavage luciferase reporter assay used as a measure of endogenous γ-secretase activity. Mean RLU ± S.E. of 5–8 independent experiments. Human BACE1 promoter activity detected by luciferase reporter. Mean RLU ± S.E. of 4–7 independent experiments. BACE1 protein levels determined by western blotting. Mean BACE1 OD: tubulin OD ± S.E. of 5–12 independent experiments.

* p < 0.05

** p < 0.01; individual comparisons by Student’s t-test with αS v1.

# p < 0.05, comparison between S129A and S129D mutants by Student’s t-test.

Amyloidogenic processing of APP appeared significantly elevated in other mutant lines without altering β-amyloid secretion. Δ71–82 caused a three-fold increase in APP-Gal4 cleavage, and nearly doubled BACE1 promoter activity, with a slight increase in BACE1 protein levels. Unlike the Δ2–9 truncation, Δ71–82 did not increase γ-secretase activity. The disease-associated point mutations A53T and A30P caused a doubling of APP-Gal4 cleavage, but did not have a clear impact on γ-secretase activity or BACE1 expression.

Interestingly, amyloidogenic processing of APP was significantly reduced in S129A SH-SY5Ys. The S129D point mutation had distinctly higher amyloidogenic processing than S129A, although not significantly greater than wildtype α-synuclein cells. Other parameters were not measured for these lines apart from BACE1 expression, which was similar in S129A to S129D.

### Iron homeostasis may link α-synuclein to enhanced APP processing and β-amyloid secretion

**Fig 7:**
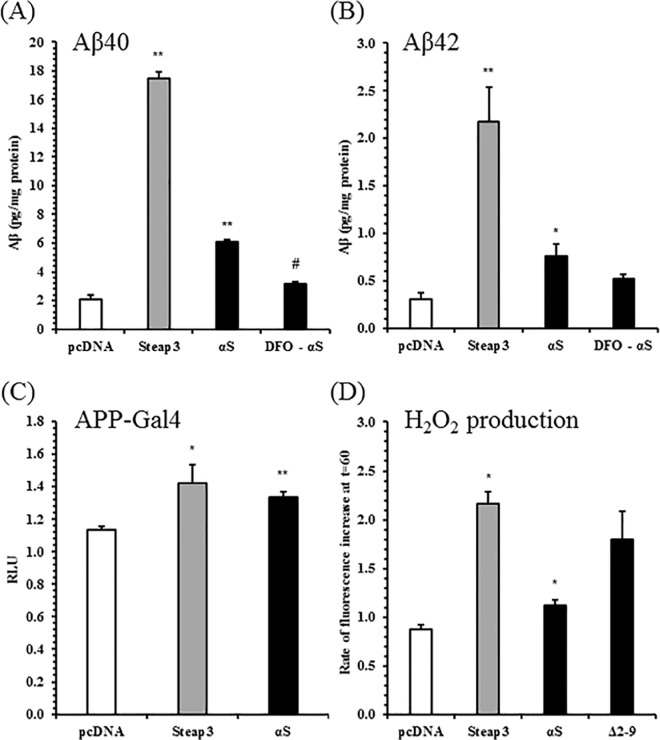
Ferrireductase Overexpression Increases β-Amyloid Secretion, APP Amyloidogenic Processing, and Oxidative Stress in SH-SY5Ys. SH-SY5Ys were stably transfected with Steap3 plasmid, and expression confirmed (data not shown). (*A)*, (*B)*, Levels of secreted Aβ40 and Aβ42 in conditioned media, ± 24 hours iron chelation, were detected with the V-PLEX Plus Aβ Peptide Panel 1 (6E10) Kit from Meso Scale Discovery. DFO: iron chelator deferoxamine, 5 μM. Peptide concentrations (pg/ml) were normalised to the total protein concentration for each sample. Mean ± S.E. of 4 biological replicates. *(C)* APP-Gal4 luciferase reporter assay, as previously described. Relative Luciferase Units (RLU) normalised to another empty vector SH-SY5Y line. Mean RLU ± S.E. of 4 independent experiments. (D) Rate of hydrogen peroxide production measured in live cells using a fluorometric CM-H_2_DCFDA probe, up to 60 minutes. Mean ± S.E. of 4 independent experiments. *, p < 0.05, **, p < 0.01 relative to empty vector; # p < 0.01 relative to other untreated αS; Student’s t-tests.

Having established that α-synuclein promotes amyloidogenic processing of APP, the underlying mechanism became a point of focus. One proposed mechanism was the putative ferrireductase activity of α-synuclein. α-Synuclein has been previously shown to reduce Fe(III) to Fe(II) in vitro, and α-syn cells have a higher ratio of Fe(II): Fe(III) [45]. It was hypothesised that an excess of free reduced iron could increase APP amyloidogenic processing. To investigate this, α-syn cells were incubated with the iron chelator deferoxamine mesylate (DFO), prior to testing secreted β-amyloid levels (Fig 7*A and* 7*B*). Aβ40 and Aβ42 levels were significantly higher in the α-syn cells than pcDNA, confirming previous data. DFO treatment attenuated Aβ40 levels in the α-syn cells, but Aβ42 levels were not significantly diminished. Additionally, we overexpressed Steap3 (S9 Fig), a potent and well-characterised ferrireductase, in SH-SY5Ys and measured secreted β-amyloid levels (Fig 7*A and* 7*B*), and APP-Gal4 cleavage activity (Fig 7*C*). Steap3 expression strongly increased β-amyloid production. Secretion of Aβ40 increased approximately 9-fold, and Aβ42 about 7-fold. Steap3 also increased β-/γ-secretase processing of the APP-Gal4 luciferase reporter.

Oxidative stress has well-established links with APP amyloidogenic processing and β-amyloid clearance. Increased levels of Fe (II) are likely to result in oxidative stress, which may mediate higher amyloidogenic processing. Oxidative stress was measured in α-synuclein and Steap3 SH-SY5Ys, to identify whether it is a likely mediator. The fluorescent probe CM-H_2_DCFDA was used to monitor the basal rate of intracellular hydrogen peroxide production over an hour in cells (Fig 7*D*). Cells overexpressing α-synuclein or Steap3 had a significantly faster rate of hydrogen peroxide production compared with empty vector cells. For α-synuclein the rate was 1.3 times faster, and for Steap3 the rate was 2.5 times faster. Cells overexpressing Δ2–9 α-synuclein were also included, but did not have a significantly higher rate of ROS production than wildtype α-synuclein SH-SY5Ys.

## Discussion

Overlap between α-synuclein and β-amyloid pathology occurs with striking frequency in neurodegenerative disease, but the molecular basis for this has not been satisfactorily explained. We have confirmed that α-synuclein stable overexpression in cells enhances β-amyloid production. Furthermore, we have shown that secretase-mediated processing of APP, but not APP expression, is upregulated as a result of increased α-synuclein. Two neuronal cell lines, human SH-SY5Y and mouse N2A, exhibited an α-synuclein-mediated induction of APP amyloidogenic processing. There was no evidence of higher γ-secretase activity, studied by a luciferase reporter method. Higher β-secretase-mediated APP processing, suggested by a greater ratio of β-CTF: full-length APP, corresponded with an increase in BACE1 protein expression in human neuroblastoma cells. BACE1 promoter activity was reduced by α-synuclein overexpression, in contrast. This phenomenon has been previously observed in cell models with transgenic prion protein expression [53] or nutrient deprivation [57], and could reflect a form of negative feedback. α-Synuclein overexpression in rat striatum also coincided with elevated BACE1 levels. We therefore propose a novel metabolic interaction between α-synuclein and APP that partly involves the specific induction of β-secretase.

Mutant α-synuclein cell lines enabled examination of the properties of α-synuclein that mediate changes to APP processing. Disease-associated point mutations, A30P, E46K, and A53T, increase the tendency of α-synuclein to form toxic oligomers [58]. The results suggest that aggregation of α-synuclein is not critical for the induction of APP amyloidogenic processing. Only E46K, but not A30P or A53T, increased β-amyloid secretion from SH-SY5Ys. Literature suggests that E46K and A53T mutations should have similar impact on α-synuclein aggregation and toxicity [59], and neither appear to affect normal function [45,60]. The elevated β-amyloid secretion in E46K cells could be an artefact of particularly robust α-synuclein expression. Markedly stronger potentiation of β-amyloid production was achieved by Δ2–9 α-synuclein expression. Δ2–9 α-synuclein is aggregation-resistant [48]. Given that aggregation-resistant α-synuclein appears to increase β-amyloid secretion by more than aggregation-prone α-synuclein, it is not likely that toxic aggregates are an important feature. Furthermore, the Δ71–82 α-synuclein cells, missing much of the β-sheet-forming ‘NAC’ region of α-synuclein, showed increased amyloidogenic processing of APP. Supporting an aggregation-independent effect, Kazmierczak et al. found that β-amyloid levels increase in PC12 cultures treated with unaggregated α-synuclein protein [32].

Data from the mutant α-synuclein cell lines suggests that the effect of α-synuclein upon APP processing increases when its physiological activities are disrupted. N-terminus truncation had the greatest impact, although it is not clear exactly how Δ2–9 affects the physiological function of α-synuclein. Previously it was proposed that loss of the N-terminal 9–11 residues abolishes membrane-binding, shown *in vitro* and in yeast, but this is not evident in neuronal cells [48,60–63]. However, the synaptic targeting of Δ2–9 α-synuclein may be mildly impaired [60]. This could trigger a loss or gain of a protein-protein interaction involving α-synuclein. Whatever the effect of Δ2–9 on α-synuclein function, Δ71–82 is likely to act similarly. Both Δ2–9 and Δ71–82 α-synuclein cells induced BACE1 promoter activation, a feature absent in all other α-synuclein SH-SY5Y lines studied. Phosphorylation mutants of α-synuclein also offer an insight into the effect of α-synuclein on APP. Cells overexpressing an S129 non-phosphorylatable mutant (S129A) appeared to have reduced APP amyloidogenic processing, relative to wildtype α-synuclein cells. An S129 phospho-mimic mutant (S129D) cell line also had significantly higher amyloidogenic processing than S129A, although not compared with the wildtype. Under physiological conditions S129 phosphorylation occurs at low levels, but is estimated to occur on approximately 90% of α-syn in Lewy bodies of DLB brains [64]. The role of S129 phosphorylation in disease progression is controversial and may be model-specific [65–71]. Functionally, the modification affects the subcellular localisation of α-synuclein, its affinity for Cu^2+^ and Fe^2+^, its propensity to aggregate, and its association with membranes [72]. The physiological role is unclear, but one proposal is that S129 phosphorylation promotes the turnover of α-synuclein through autophagy [73,74]. A role in synaptic transmission has also been suggested [72]. A functional proteomics study revealed that phosphorylated α-synuclein has greater affinity for proteins involved in synaptic transmission and vesicle trafficking [75]. Rab8a was later confirmed to bind to α-synuclein in an S129 phosphorylation-dependent manner [76].

α-Synuclein may co-localise with APP, since APP has been found in pre-synaptic vesicles, a major site of α-synuclein membrane binding [77–79]. Yet to alter the amyloidogenic processing of APP it is not necessary for α-synuclein to be in close proximity, since α-synuclein overexpression is known to profoundly affect the secretory pathway in cell models [80,81]. This could change the subcellular localisation or turnover of APP and the secretases. Amyloidogenic processing of APP and β-amyloid production are also induced by stress signalling from metals, oxidative stress, excitotoxicity, neuroinflammation, or hypercholesterolaemia [82]. Downstream jun kinase (JNK), peroxisome proliferator-activated receptor γ (PPAR-γ), nuclear factor-κB (NF-κB), or nuclear factor of activated T-cells (NFAT) signalling can transcriptionally activate β- and γ-secretase production [36,37,83–85]. Calcium stress, oxidative stress, ER stress, and nutrient deprivation also activate pathways that converge on eIF2α, resulting in translational induction of the β-secretase BACE1 [38,57,86]. It is likely that α-synuclein activates stress signalling in the cell through one or more of the aforementioned factors.

Identifying the precise connection between α-synuclein and APP secretase-mediated processing would be invaluable. We propose that α-synuclein-induced oxidative stress, potentially connected to its activity as a putative ferrireductase [45], could play a role. Higher levels of reduced iron can be measured in SH-SY5Ys stably overexpressing α-synuclein [45]. Iron has the effect of both promoting APP translation, and APP amyloidogenic processing [42–44,87]. In the present study the ferrireductase enzyme Steap3, and putative ferrireductase α-synuclein, were both associated with high APP amyloidogenic processing and β-amyloid production in neuroblastoma cells. Levels of intracellular iron accumulation were not directly compared between the α-synuclein and Steap3 cells. However, a role for iron in α-synuclein-induced β-amyloid production is supported by attenuation with an iron chelator. The same iron chelator has been previously shown to decrease β-amyloid deposition in a rodent model [42]. Oxidative stress could potentially be involved since basal ROS generation was significantly elevated in both α-synuclein and Steap3 cells. Our finding agrees with previous work showing that α-synuclein increases oxidative stress in cells [88–90]. Oxidative stress affects amyloidogenic APP processing through several pathways. Studies have shown that oxidative stimuli promote JNK- or NF-κB-mediated BACE1 transcription, which may be tightly linked to γ-secretase activity [35–37]. BACE1 translation may also be upregulated by oxidative stress via protein kinase R (PKR), which promotes eIF2α-mediated translational de-repression, and also activates JNK [38]. Altered subcellular localisation of BACE1 in response to oxidative stress has also been proposed to increase APP amyloidogenic processing [39]. As a potential mediator of the novel metabolic interaction between α-synuclein and APP, oxidative stress is worth further investigation.

In recent years, a developing body of literature has documented the association between biomarkers of α-synuclein and β-amyloid pathology in neurodegenerative disease [91]. Little progress has been made on the molecular details of this association. A particularly strong association between α-synuclein and β-amyloid pathology exists in DLB, which comprises 4–8% of all dementia diagnoses yet is relatively poorly understood [92]. We hope that the demonstration that α-synuclein can promote β-secretase-mediated processing of APP in cells will stimulate further mechanistic research. Detailing the underlying molecular mechanism is of primary importance, and could lead to new avenues for therapeutics. In particular it is important to understand the species of α-synuclein that trigger changes to APP processing. Whether or not α-synuclein aggregates are involved could have implications for the success of aggregate-targeting immunotherapies in development [93].

In summary, β-secretase-mediated processing of APP may be potentiated by high levels of α-synuclein, and could represent a contributing mechanism of β-amyloid accumulation in synucleinopathy disease.

## Supporting information

S1 Fig Expression of α-Synuclein in Wildtype α-Synuclein Overexpressing SH-SY5Ys. SH-SY5Ys were stably transfected with wildtype α-synuclein expression plasmid to generate three independent lines. Western blotting confirms α-synuclein overexpression. Raw band optical intensities (OD) expressed as a ratio of α-synuclein to α-tubulin. Mean ± S.E. of 5 independent experiments. ** p < 0.01 relative to empty vector; Student’s t-tests.
(TIF)

S2 Fig Validation of APP-Gal4 and Notch-Gal4 Luciferase Reporters. Compound treatment 6 hours post-transfection, and luciferase readout performed after 16 hours as detailed in the Experimental Procedures. TAPI-1 (50 μM) was used to inhibit α-secretase, ‘βSI’ and ‘β-IV’ (10 μM) were used to inhibit β-secretase, and DAPT (10 μM) was used to inhibit γ-secretase. *(A)* The APP-Gal4 assay preferentially reports β-/γ-secretase-mediated processing. *(B)* The Notch-Gal4 assay in αS v1 cells reports γ-secretase-mediated processing. Mean ± S.E. of a minimum of 3 independent experiments. **, p < 0.01 relative to pLuc +APP-Gal4; one-way ANOVA with Tukey post-hoc test. RLU: Relative Luciferase Units.
(TIF)

S3 Fig Expression of α-Synuclein in α-Synuclein Overexpressing N2As. N2As were stably transfected with wildtype α-synuclein expression plasmid. Western blotting confirms α-synuclein overexpression. Raw band optical intensities (OD) expressed as a ratio of α-synuclein to α-tubulin. Mean ± S.E. of 5 independent experiments. ** p < 0.01 relative to empty vector; pairwise t-tests with a Holm adjustment.
(TIF)

S4 Fig Increased APP Amyloidogenic Processing in α-Synuclein Overexpressing N2As. APP-Gal4 activity in α-synuclein N2A cells. Mean ± S.E. of 5 independent experiments. ** p < 0.01 relative to pcDNA; Student’s t-test. RLU: Relative Luciferase Units.
(TIF)

S5 Fig Accumulation of APP C-Terminal Fragments (CTFs) with γ-Secretase Inhibition. Western blot for APP CTFs in pcDNA and α-synuclein SH-SY5Ys incubated with 2 μM DAPT, compared with untreated pcDNA SH-SY5Ys. Dotted line indicates a break in a single blot.
(TIF)

S6 Fig Expression and Aggregation of Overexpressed Human α-Synuclein in the Substantia Nigra and Striatum of Rats Unilaterally Injected with AAV6-α-syn. (*A*) Co-immunostaining for α-synuclein (green) and aggregated α-synuclein (5G4 antibody, red) in the substantia nigra of an AAV6-α-syn injected rat. The absence of signal in the substantia nigra of a rat injected with a non-coding AAV6 vector demonstrates the specificity of the immunostaining. (*B*) Co-immunostaining for α-synuclein (green) and aggregated α-synuclein (5G4 antibody, red) in the striatum of an AAV6-α-syn injected rat. The absence of signal in the striatum of a rat injected with a non-coding AAV6 vector demonstrates the specificity of the immunostaining. Note the presence of aggregated α-synuclein (5G4 positive) both in the substantia nigra (mainly in neuronal soma) and in the striatum (mainly axonal) of AAV6-α-syn injected animals. (*C*) Striatal section immunostained for tyrosine hydroxylase (TH). Note the loss of TH immunoreactivity in the hemisphere injected with the AAV6-α-syn vector (indicated by *), which shows mild neurodegeneration at three months post-injection.
(TIF)

S7 Fig Expression of Striatal BACE1 in Empty AAV6 Vector-Injected Rats is Unaltered. Western blot of BACE1 and α-tubulin expression in striata from four empty AAV6 vector-injected rats, contralateral (“Con”) and ipsilateral (“Inf”) to vector injection, quantified in Fig 6.
(TIF)

S8 Fig Expression of α-Synuclein in Mutant α-Synuclein Overexpressing SH-SY5Ys. α-Synuclein overexpression confirmed by western blotting. Raw band optical intensities (OD) expressed as a ratio of α-synuclein to α-tubulin. Mean ± S.E. of 7 independent experiments. No significant differences between α-synuclein lines; pairwise t-tests with a Holm adjustment.
(TIF)

S9 Fig Expression of Steap3 in Steap3 Overexpressing SH-SY5Ys. Western blotting confirms Steap3 overexpression.
(TIF)
