# Supplementary figures and images for: α-Synuclein increases β-amyloid secretion by promoting β-/γ-secretase processing of APP
Source: PLoS One. 2017 Feb 10;12(2):e0171925. doi: 10.1371/journal.pone.0171925 (PMC5302447; doi:10.1371/journal.pone.0171925)

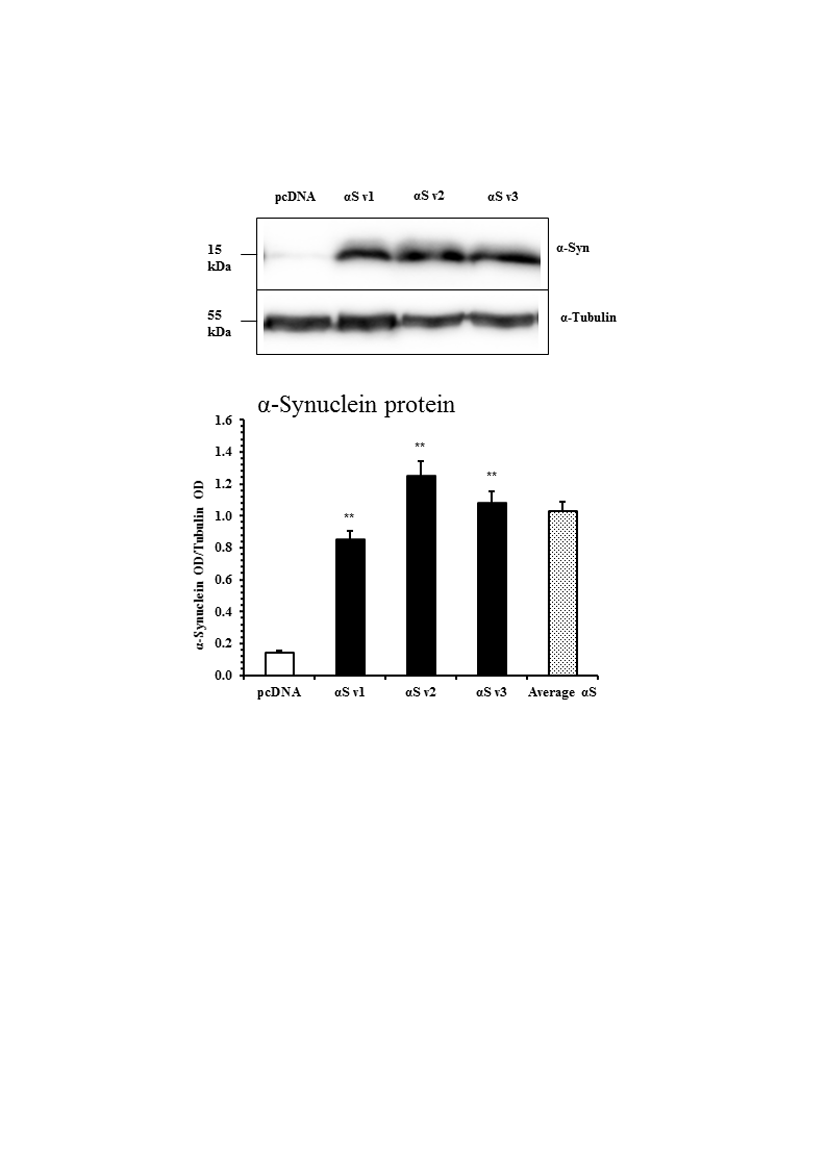

Supplement: S1 Fig — SH-SY5Ys were stably transfected with wildtype α-synuclein expression plasmid to generate three independent lines. Western blotting confirms α-synuclein overexpression. Raw band optical intensities (OD) expressed as a ratio of α-synuclein to α-tubulin. Mean ± S.E. of 5 independent experiments. ** p < 0.01 relative to empty vector; Student’s t-tests. (TIF) [file pone.0171925.s001.tif]

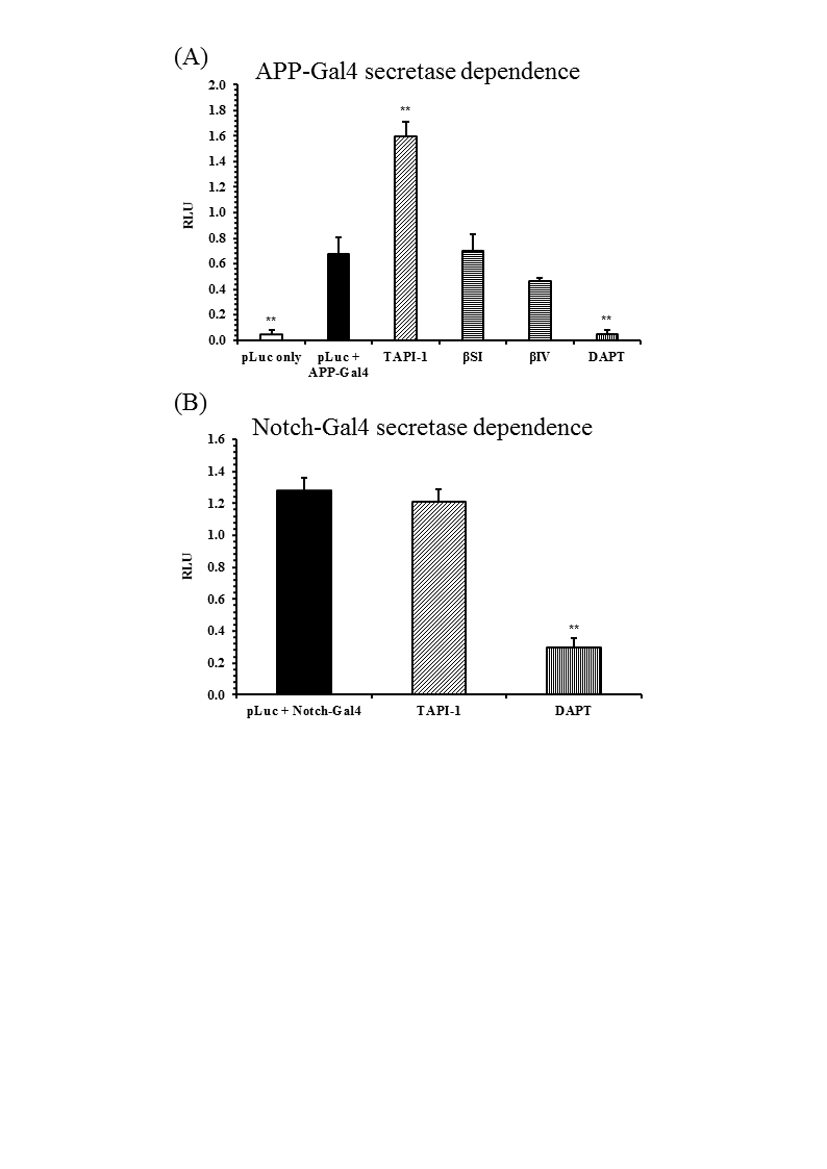

Supplement: S2 Fig — Compound treatment 6 hours post-transfection, and luciferase readout performed after 16 hours as detailed in the Experimental Procedures. TAPI-1 (50 μM) was used to inhibit α-secretase, ‘βSI’ and ‘β-IV’ (10 μM) were used to inhibit β-secretase, and DAPT (10 μM) was used to inhibit γ-secretase. (A) The APP-Gal4 assay preferentially reports β-/γ-secretase-mediated processing. (B) The Notch-Gal4 assay in αS v1 cells reports γ-secretase-mediated processing. Mean ± S.E. of a minimum of 3 independent experiments. **, p < 0.01 relative to pLuc +APP-Gal4; one-way ANOVA with Tukey post-hoc test. RLU: Relative Luciferase Units. (TIF) [file pone.0171925.s002.tif]

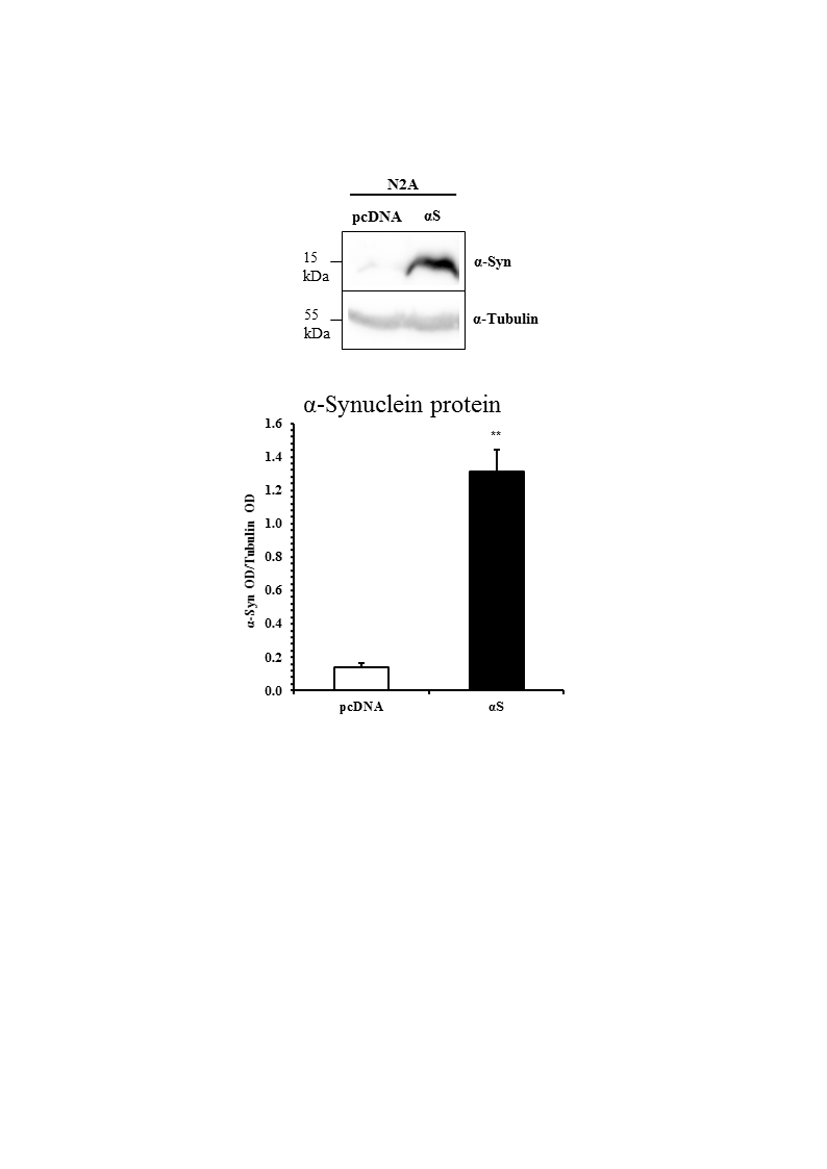

Supplement: S3 Fig — N2As were stably transfected with wildtype α-synuclein expression plasmid. Western blotting confirms α-synuclein overexpression. Raw band optical intensities (OD) expressed as a ratio of α-synuclein to α-tubulin. Mean ± S.E. of 5 independent experiments. ** p < 0.01 relative to empty vector; pairwise t-tests with a Holm adjustment. (TIF) [file pone.0171925.s003.tif]

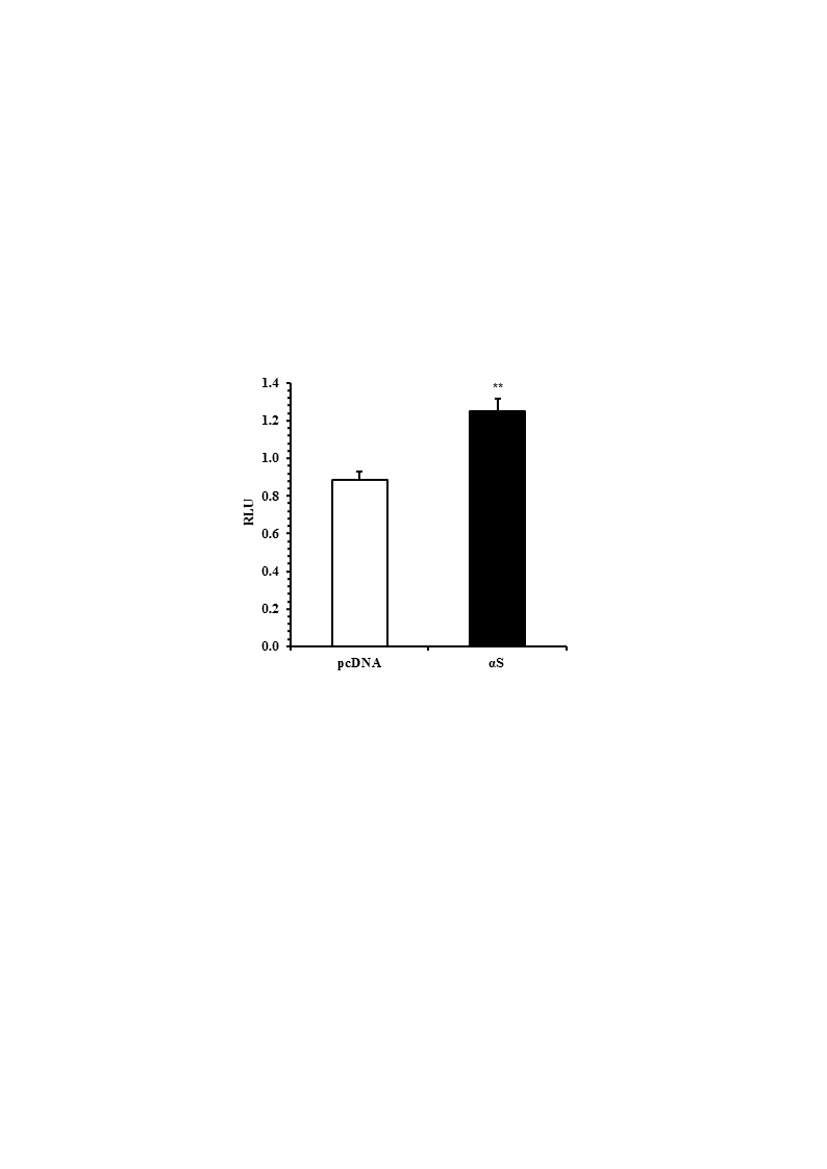

Supplement: S4 Fig — APP-Gal4 activity in α-synuclein N2A cells. Mean ± S.E. of 5 independent experiments. ** p < 0.01 relative to pcDNA; Student’s t-test. RLU: Relative Luciferase Units. (TIF) [file pone.0171925.s004.tif]

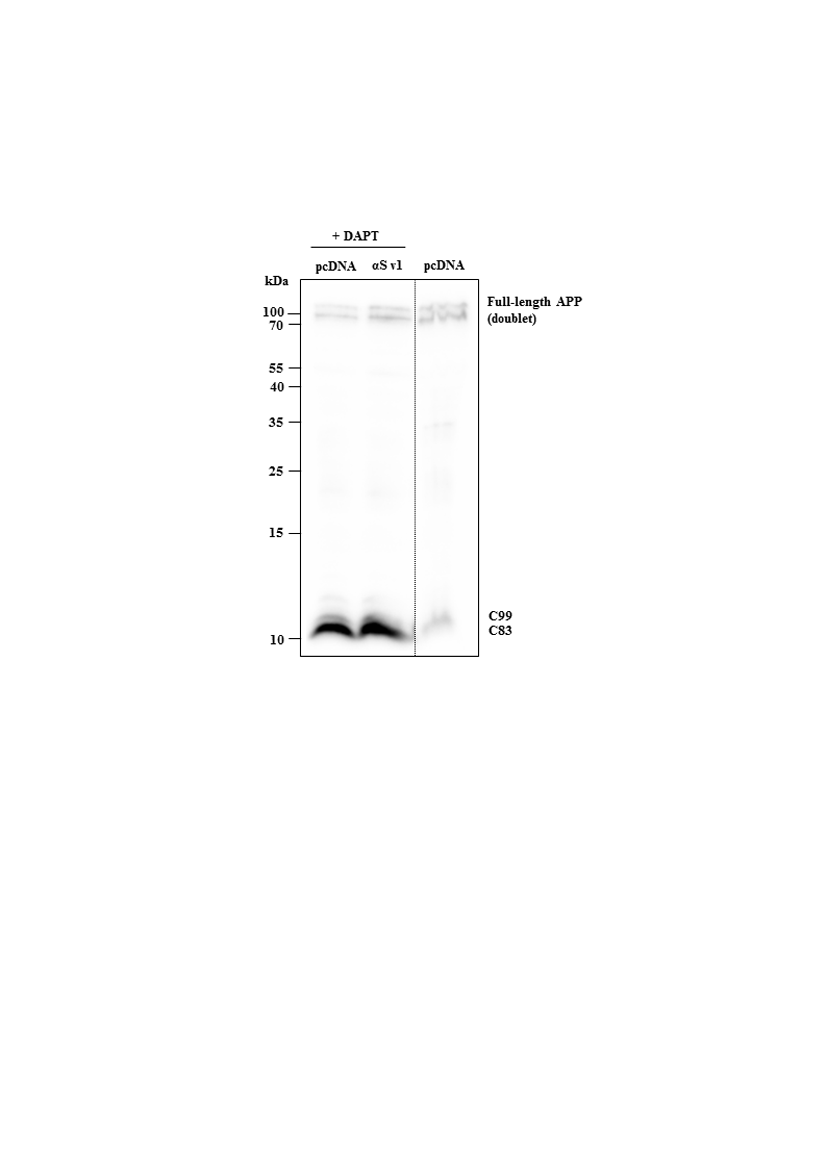

Supplement: S5 Fig — Western blot for APP CTFs in pcDNA and α-synuclein SH-SY5Ys incubated with 2 μM DAPT, compared with untreated pcDNA SH-SY5Ys. Dotted line indicates a break in a single blot. (TIF) [file pone.0171925.s005.tif]

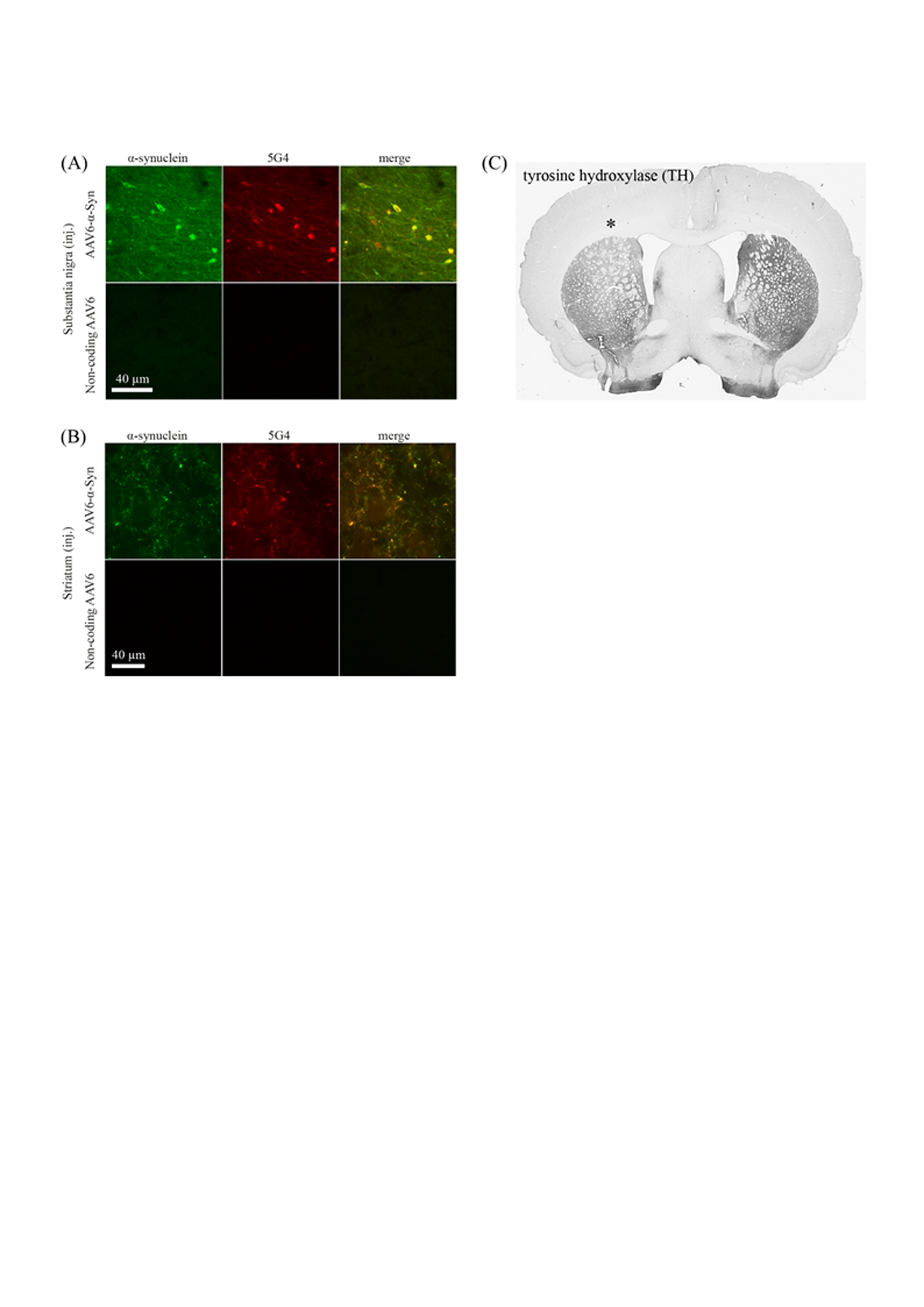

Supplement: S6 Fig — (A) Co-immunostaining for α-synuclein (green) and aggregated α-synuclein (5G4 antibody, red) in the substantia nigra of an AAV6-α-syn injected rat. The absence of signal in the substantia nigra of a rat injected with a non-coding AAV6 vector demonstrates the specificity of the immunostaining. (B) Co-immunostaining for α-synuclein (green) and aggregated α-synuclein (5G4 antibody, red) in the striatum of an AAV6-α-syn injected rat. The absence of signal in the striatum of a rat injected with a non-coding AAV6 vector demonstrates the specificity of the immunostaining. Note the presence of aggregated α-synuclein (5G4 positive) both in the substantia nigra (mainly in neuronal soma) and in the striatum (mainly axonal) of AAV6-α-syn injected animals. (C) Striatal section immunostained for tyrosine hydroxylase (TH). Note the loss of TH immunoreactivity in the hemisphere injected with the AAV6-α-syn vector (indicated by *), which shows mild neurodegeneration at three months post-injection. (TIF) [file pone.0171925.s006.tif]

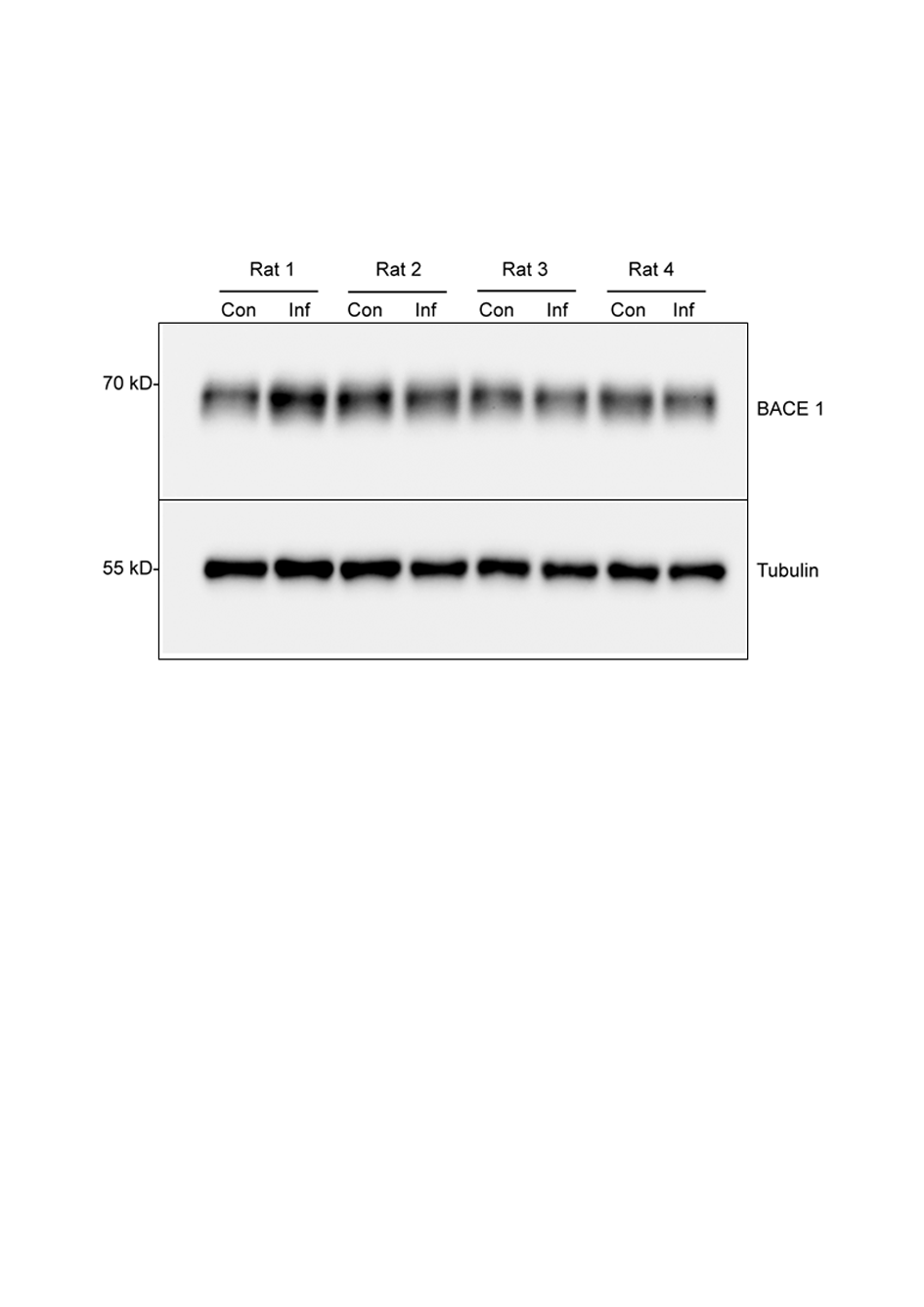

Supplement: S7 Fig — Western blot of BACE1 and α-tubulin expression in striata from four empty AAV6 vector-injected rats, contralateral (“Con”) and ipsilateral (“Inf”) to vector injection, quantified in Fig 6. (TIF) [file pone.0171925.s007.tif]

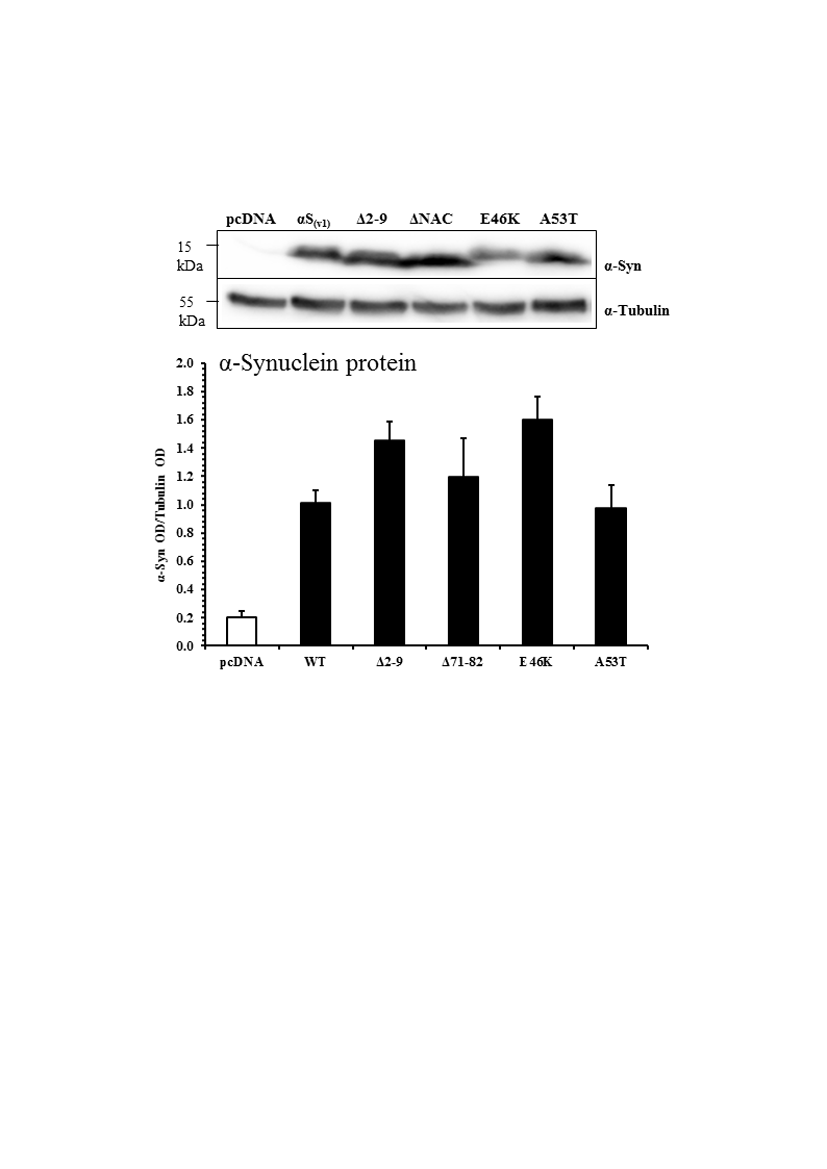

Supplement: S8 Fig — α-Synuclein overexpression confirmed by western blotting. Raw band optical intensities (OD) expressed as a ratio of α-synuclein to α-tubulin. Mean ± S.E. of 7 independent experiments. No significant differences between α-synuclein lines; pairwise t-tests with a Holm adjustment. (TIF) [file pone.0171925.s008.tif]

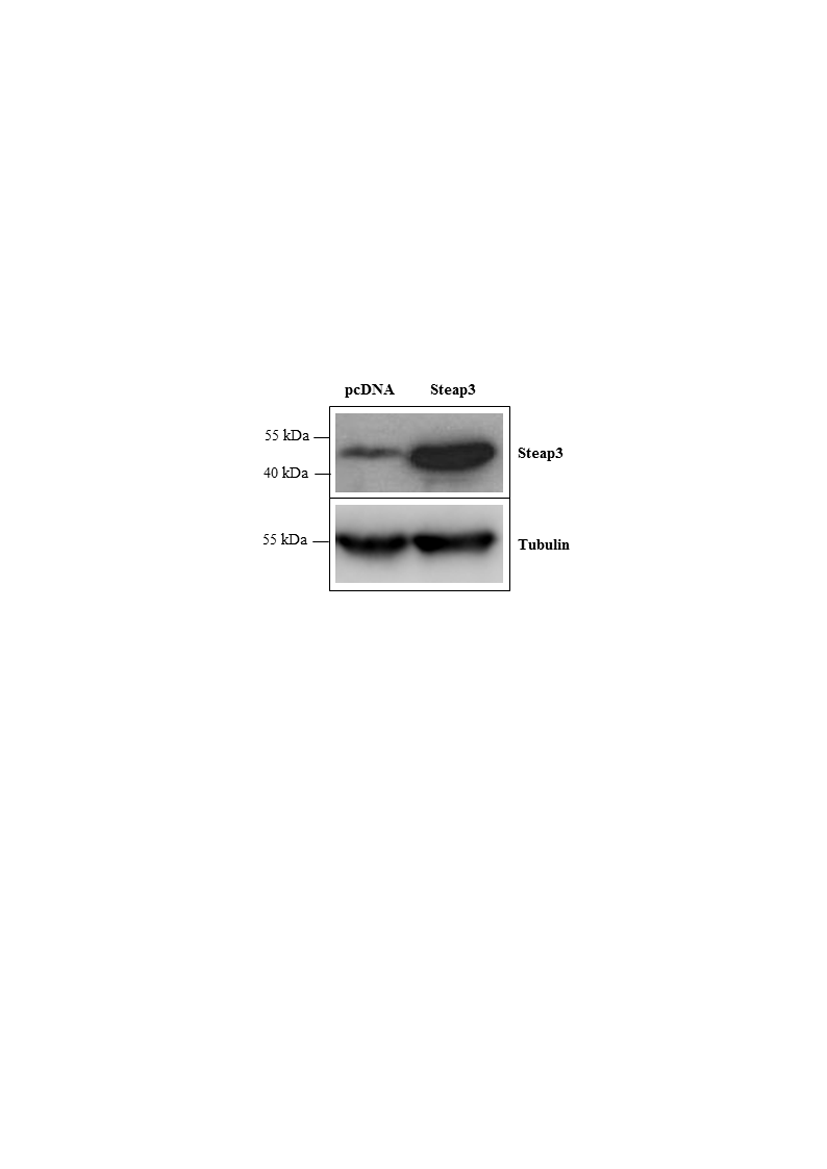

Supplement: S9 Fig — Western blotting confirms Steap3 overexpression. (TIF) [file pone.0171925.s009.tif]
